# Near-real-time estimation of fossil fuel CO_2_ emissions from China based on atmospheric observations on Hateruma and Yonaguni Islands, Japan

**DOI:** 10.1186/s40645-023-00542-6

**Published:** 2023-03-02

**Authors:** Yasunori Tohjima, Yosuke Niwa, Prabir K. Patra, Hitoshi Mukai, Toshinobu Machida, Motoki Sasakawa, Kazuhiro Tsuboi, Kazuyuki Saito, Akihiko Ito

**Affiliations:** 1 National Institute for Environmental Studies (NIES), 16-2 Onogawa, Tsukuba, Ibaraki 305-8506 Japan; 2 Japan Agency for Marine-Earth Science and Technology (JAMSTEC), 3173-25 Showa-Machi, Kanazawa-Ku, Yokohama, Kanagawa 236-0001 Japan; 3 Meteorological Research Institute (MRI), 1-1 Nagamine, Tsukuba, Ibaraki 305-0052 Japan; 4 Japan Meteorological Agency (JMA), 3-6-9 Toranomon, Minato-Ku, Tokyo, 105-8431 Japan

**Keywords:** Fossil fuel CO_2_ emissions, Synoptic-scale variations, Atmospheric CO_2_, Atmospheric CH_4_, COVID-19 lockdown, East Asian Monsoon

## Abstract

We developed a near-real-time estimation method for temporal changes in fossil fuel CO_2_ (FFCO_2_) emissions from China for 3 months [January, February, March (JFM)] based on atmospheric CO_2_ and CH_4_ observations on Hateruma Island (HAT, 24.06° N, 123.81° E) and Yonaguni Island (YON, 24.47° N, 123.01° E), Japan. These two remote islands are in the downwind region of continental East Asia during winter because of the East Asian monsoon. Previous studies have revealed that monthly averages of synoptic-scale variability ratios of atmospheric CO_2_ and CH_4_ (ΔCO_2_/ΔCH_4_) observed at HAT and YON in JFM are sensitive to changes in continental emissions. From the analysis based on an atmospheric transport model with all components of CO_2_ and CH_4_ fluxes, we found that the ΔCO_2_/ΔCH_4_ ratio was linearly related to the FFCO_2_/CH_4_ emission ratio in China because calculating the variability ratio canceled out the transport influences. Using the simulated linear relationship, we converted the observed ΔCO_2_/ΔCH_4_ ratios into FFCO_2_/CH_4_ emission ratios in China. The change rates of the emission ratios for 2020–2022 were calculated relative to those for the preceding 9-year period (2011–2019), during which relatively stable ΔCO_2_/ΔCH_4_ ratios were observed. These changes in the emission ratios can be read as FFCO_2_ emission changes under the assumption of no interannual variations in CH_4_ emissions and biospheric CO_2_ fluxes for JFM. The resulting average changes in the FFCO_2_ emissions in January, February, and March 2020 were 17 ± 8%, − 36 ± 7%, and − 12 ± 8%, respectively, (− 10 ± 9% for JFM overall) relative to 2011–2019. These results were generally consistent with previous estimates. The emission changes for January, February, and March were 18 ± 8%, − 2 ± 10%, and 29 ± 12%, respectively, in 2021 (15 ± 10% for JFM overall) and 20 ± 9%, − 3 ± 10%, and − 10 ± 9%, respectively, in 2022 (2 ± 9% for JFM overall). These results suggest that the FFCO_2_ emissions from China rebounded to the normal level or set a new high record in early 2021 after a reduction during the COVID-19 lockdown. In addition, the estimated reduction in March 2022 might be attributed to the influence of a new wave of COVID-19 infections in Shanghai.

**Supplementary Information:**

The online version contains supplementary material available at 10.1186/s40645-023-00542-6.

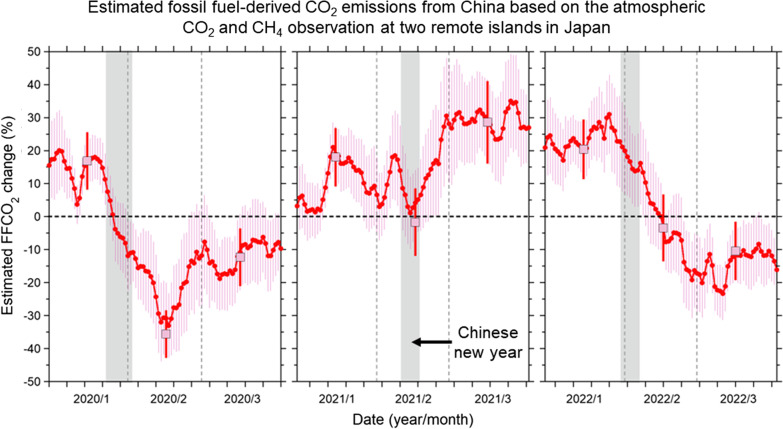

## Introduction

To curb the impacts of global warming, a legally binding international treaty, the Paris Agreement, was adopted at the UN Climate Change Conference (COP21) in Paris on December 13, 2015. The goal of the Paris Agreement is to limit the temperature rise to well less than 2 °C, preferably 1.5 °C, in comparison with pre-industrial levels. The implementation of the agreement requires a worldwide effort to immediately reduce emissions of anthropogenic greenhouse gases (GHGs) (United Nations Environment Programme 2021). The steady implementation of the GHGs reductions pledged by individual countries requires the development of validation methods for regional/country-scale GHGs emissions. The national carbon dioxide (CO_2_) emissions from fossil fuel combustion and cement manufacture (FFCO_2_) are usually derived from inventories based on various statistical data, including the production, consumption, and trade of fossil fuels (e.g., Gilfillan and Marland 2021). However, it is also crucial to develop independent observational methods for the estimation of emissions for tracking policy implementation.

Systematic observations of atmospheric GHGs, including CO_2_ and methane (CH_4_), have been conducted by many laboratories around the world. Even in the East Asian region, intensive GHGs observation networks have been developed using different platforms, such as ground-based stations (e.g., Tsutsumi et al. 2006; Tohjima et al. 2014), ships (e.g., Terao et al. 2011; Tohjima et al. 2012), aircraft (e.g., Machida et al. 2008; Tsuboi et al. 2013; Umezawa et al. 2020), and satellites (e.g., Yokota et al. 2009; Yoshida et al. 2013). There are now denser networks of atmospheric observations around the globe than before. Meanwhile, the objective of the atmospheric observations has been extended from clarification of the global trends and temporal changes in the atmospheric burdens to the quantitative evaluation of regional/country-scale fluxes with the help of atmospheric transport models.

In these circumstances, a global pandemic of the novel coronavirus disease, COVID-19, broke out in early 2020, and severe actions restricting socioeconomic activity, including city lockdowns, were imposed by the concerned countries to prevent the spread of COVID-19. These actions were also expected to decrease fossil fuel consumption, resulting in a reduction in the emissions of related species, including NO_*x*_, CO_2_, and so on. For example, ground-based and satellite-based observations revealed that atmospheric NO_2_ concentrations decreased by 10–70% over the cities in East China during the lockdown period from late January to March 2020 compared with those in 2019 (e.g., Bauwens et al. 2020; Le et al. 2020). Using such satellite-based column-averaged NO_2_ distributions, models, and a variety of bottom-up information, Zheng et al. (2020) estimated an 11.5% decrease in China’s CO_2_ emissions during January–April 2020 compared to the same period in 2019. Meanwhile, the studies on changes in CO_2_ emissions during the COVID-19 period were conducted based on various activity data: the change in FFCO_2_ emissions from China estimated by Le Quéré et al. (2020) was − 242 (− 108 to − 394) MtCO_2_ during January–April 2020, which corresponds to a − 6.9% (− 3.1 to − 11.2%) decrease compared with the emissions during the same period in 2019. Such activity data-based estimates allow us to evaluate the detailed temporal change. For example, Liu et al. (2020) reported that the changes in the monthly FFCO_2_ emissions in 2020 from 2019 were − 18.4% in February, − 9.2% in March, and + 0.6% in April. These results raised the question of whether direct observations of atmospheric CO_2_ were able to detect the signals related to the FFCO_2_ emission reduction caused by the COVID-19 outbreak.

Short-lived pollution constituents like NO_*x*_, whose estimated lifetime over China is less than a day even in winter when NO_*x*_ has the longest lifetime (Shah et al. 2020), showed considerable decreases associated with the COVID-19 lockdown in China, as mentioned above. In contrast, the change in the atmospheric CO_2_ mole fraction caused by the COVID-19 pandemic is considered to be relatively small in comparison with the atmospheric CO_2_ level because of its relatively large amount in the atmosphere and a rather long lifetime. The estimated decrease in the annual global FFCO_2_ emissions in 2020 was 5–7% relative to that in 2019, which was about 10 PgC (Le Quéré et al. 2021; Friedlingstein, et al. 2022). Since the estimated change of 0.5–0.7 PgC corresponds to the globally averaged atmospheric CO_2_ mole fraction of 0.2–0.3 ppm, it is rather difficult to detect such subtle signals in the atmospheric CO_2_ trends after the emitted CO_2_ is mixed globally (Lovenduski et al. 2021). Nevertheless, several studies succeeded in detecting signals related to the FFCO_2_ reductions in China caused by the COVID-19 lockdown in both local-scale observations (Zeng et al. 2022; Liu et al. 2021; Wu et al. 2021) and regional-scale observations (Tojima et al. 2020; Buchwitz et al. 2021; Weir et al. 2021; Sim et al. 2022) of atmospheric CO_2_.

Tohjima et al. (2020) applied a unique method in their study, which was one of the first studies to observationally detect the regional-scale signals related to FFCO_2_ emission decreases caused by the COVID-19 lockdown in China from the synoptic-scale variability ratio of the atmospheric CO_2_ and CH_4_ (ΔCO_2_/ΔCH_4_) observed on Hateruma Island (HAT, 24.06° N, 123.81° E). Hateruma Island is located in the downwind area of continental East Asia from late autumn to early spring due to the influence of the East Asian monsoon. A previous study revealed that the ΔCO_2_/ΔCH_4_ ratio roughly reflected the emission ratio of CO_2_–CH_4_ from continental East Asia, especially China (Tohjima et al. 2014). In that study, the observed ΔCO_2_/ΔCH_4_ ratios showed a gradual increase during 2000–2010, reflecting an unprecedented increase in FFCO_2_ emissions associated with the rapid economic growth in China. The monthly mean ΔCO_2_/ΔCH_4_ ratio showed a marked decrease in February 2020 when a severe lockdown was implemented almost across China (Tohjima et al. 2020). By using the observed changes in the ΔCO_2_/ΔCH_4_ ratios and the simulated relationship between the ΔCO_2_/ΔCH_4_ ratio and the FFCO_2_ emissions from China, we estimated the FFCO_2_ reductions to be 32 ± 12% for February and 19 ± 15% for March 2020. More recently, examining the ΔCO_2_/ΔCH_4_ ratio on Yonaguni Island (YON, 24.47° N, 123.01° E), located only about 90 km northwest of HAT, we found that the ΔCO_2_/ΔCH_4_ ratio also showed a marked decrease in February 2020 after eliminating the local influences (Tohjima et al. 2022). These results convinced us of the reliability of the ΔCO_2_/ΔCH_4_ ratio as an indicator of the relative emission strength in China.

In this study, we revisited the ΔCO_2_/ΔCH_4_ ratios observed at HAT and YON to develop a near-real-time estimation method for the temporal change in the FFCO_2_ emissions from China and updated the results for 2021 and 2022. In our previous study (Tohjima et al. 2020), we used prior information about the temporal variation of the FFCO_2_ emissions based on a bottom-up estimation by Le Quéré et al. (2020) to evaluate the FFCO_2_ emission change in China in 2020. Here, we developed a method that does not need any prior information about the temporal emission changes. We examined the relationship between the ΔCO_2_/ΔCH_4_ ratio and the FFCO_2_/CH_4_ emission ratio in China by using an atmospheric transport model and including all components of the surface fluxes. Based on the simulated relationship and the ΔCO_2_/ΔCH_4_ ratios observed at HAT and YON, we estimated the FFCO_2_ emission changes in China during January–March (JFM) in 2020, 2021, and 2022 under the assumption of invariable biospheric CO_2_ fluxes and total CH_4_ emissions. These results were then compared with reported estimations for earlier times.

## Methods/experimental

### Atmospheric observations at HAT and YON

**Fig. 1:**
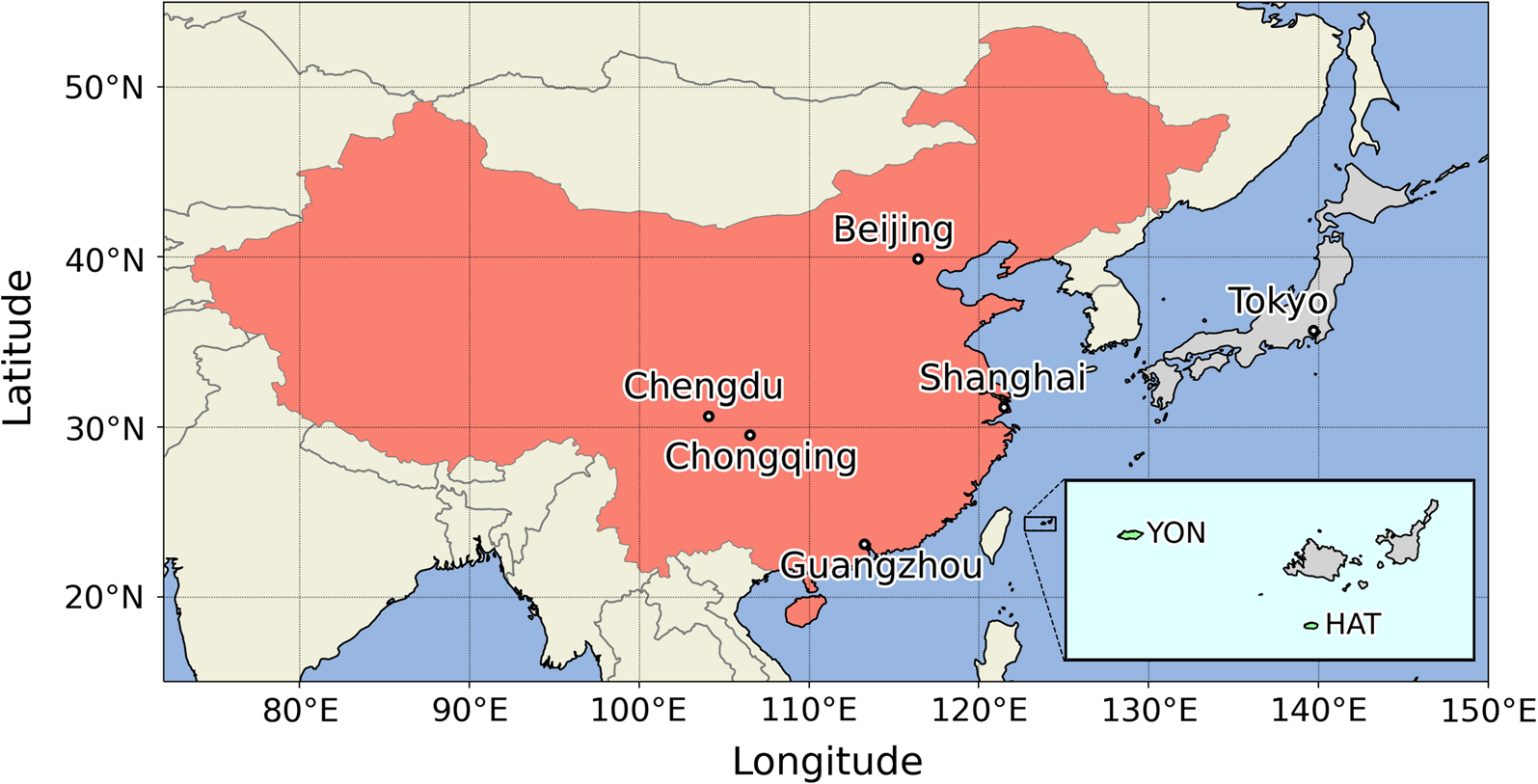
Map showing the locations of Hateruma Island (HAT) and Yonaguni Island (YON)

The National Institute for Environmental Studies (NIES) and the Japan Meteorological Agency (JMA) began monitoring the atmospheric GHGs, including CO_2_ and CH_4_ at HAT and YON, respectively, in the 1990s (Fig. 1). The technical details for the measurements of atmospheric CO_2_ and CH_4_ were given in Tohjima et al. (2002) and Tohjima et al. (2010) for HAT, and Watanabe et al. (2000) and Tsutsumi et al. (2006) for YON. Both islands belong to the western part of the Ryukyu Islands, located between the East China Sea and the western Pacific. Air masses are predominantly transported from the continental region of East Asia during winter and from the Pacific region during summer due to the East Asian monsoon (Wada et al. 2013; Tohjima et al. 2014). Since HAT and YON are relatively closely located to each other within a distance of about 90 km, almost identical seasonal cycles and trends of the atmospheric CO_2_ and CH_4_ have been observed on both islands (Zhang et al. 2007).

**Fig. 2:**
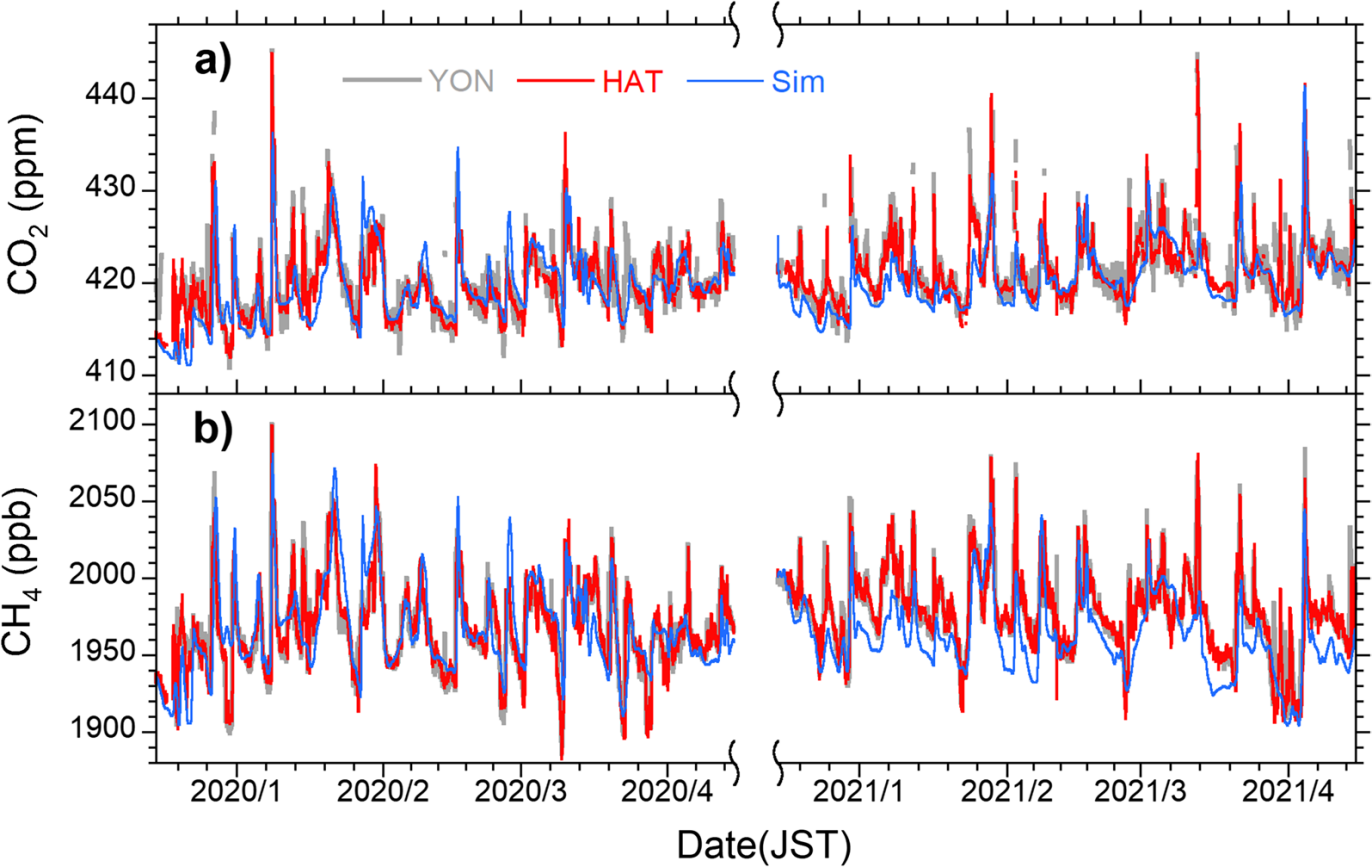
Time series of (**a**) atmospheric CO_2_ and (**b**) CH_4_ hourly mole fractions. The data obtained at YON (gray lines) and HAT (red lines) during the periods from December 15, 2019, to April 15, 2020, and from December 15, 2020, to April 15, 2021, are depicted. The simulated CO_2_ and CH_4_ for HAT are plotted as blue lines (see text)

Additionally, similar synoptic-scale variations with periods of several hours to several days have also been observed at both sites, especially during winter, as shown in Fig. 2. Enhanced mole fractions of GHGs and related species are often observed when the continental air masses are transported to the islands. However, a previous study revealed that a substantial diurnal variation was superimposed on the synoptic-scale variation of CO_2_ at YON, preventing us from extracting the continental emission signals from the variability ratio (Tohjima et al. 2022). This diurnal cycle with a deep trough in the daytime was attributed to the local biospheric CO_2_ exchange on the island. The rather large local influences at YON were attributed to the differences in the site conditions: the monitoring station with a sampling tower at HAT was built at the eastern tip of the island, whereas that at YON is located inland. We needed a different treatment for the data at YON to suppress the local influences (Tohjima et al. 2022). In this study, we used hourly CO_2_ and CH_4_ data at HAT and YON up to April 2022.

### Data processing procedure

The ratio of the synoptic-scale variations of the atmospheric CO_2_ and CH_4_ was calculated using the same methods as those adopted by previous studies (Tohjima, et al. 2014, 2020). Here, we provide a brief explanation of the calculation procedure. The variability ratio, ΔCO_2_/ΔCH_4_, was computed as a slope of reduced major axis regression (RMA) (Hirsch and Gilroy 1984) for a scatter plot of the consecutive time series of the two species within a certain time window. The standard deviations and the correlation coefficient were also computed at the same time. These calculations were repeated for the whole data set by shifting the time window by one hour. Then, if the standard deviation and the correlation coefficient were lower than certain criteria, those regression slopes were discarded. Finally, the selected regression slopes were used to compute the monthly average or the moving averages of ΔCO_2_/ΔCH_4_.

We set 0.1 ppm and 0.7 as the criteria for the standard deviation of CO_2_ and the correlation coefficient, respectively, as was done in our previous study. As for the time window for the correlation analysis, a duration of 24 h was used for HAT, while a much longer duration was used for YON. As mentioned in the previous section, the CO_2_ diurnal cycle at YON showed a larger decrease in the daytime than that at HAT, which can be attributed to a larger local CO_2_ uptake at YON. The larger diurnal cycle, enlarging the absolute value of the ΔCO_2_/ΔCH_4_ ratio at YON, made it difficult to extract the signals related to the relative emission strengths in the upwind region. However, since such a local influence was effectively eliminated by using a longer time window (84 h) and only nighttime data in a previous study (20-06 LST), we adopted the same approach as Tohjima et al. (2022) to calculate the ΔCO_2_/ΔCH_4_ ratio at YON in this study.

### Model simulation

To quantitatively evaluate the relationship between the continental CO_2_ and CH_4_ emissions and the ΔCO_2_/ΔCH_4_ ratio at HAT and YON, we used a Nonhydrostatic ICosahedral Atmospheric Model (NICAM)-based atmospheric transport model (NICAM-TM: Niwa et al. 2011). The NICAM dynamical framework inherently guarantees the conservation of tracer mass in the atmospheric transport process without any numerical mass fixer (Satoh 2002), which makes NICAM-TM suitable for studying long-lived species like greenhouse gases (e.g., Niwa et al. 2012). The Japanese 55-year Reanalysis data (JRA-55: Kobayashi et al. 2015) for the period between 2000 and 2021 were used to nudge horizontal winds in the NICAM-TM simulation, and the horizontal resolution of NICAM-TM used was approximately 112 km.

For the simulation of the atmospheric CO_2_, we used all components of the global surface CO_2_ fluxes, which consist of fluxes of FFCO_2_, ocean CO_2_, and land biosphere CO_2_ (BioCO_2_). For the FFCO_2_, we used global high-resolution flux maps from the Open-source Data Inventory for Anthropogenic CO_2_, version 2019 (ODIAC2019), which were available for the period from 2000 to 2018 (Oda and Maksyutov 2011; Oda et al. 2018). For the ocean CO_2_, we used monthly air-sea flux maps developed by the Japan Meteorological Agency for the period from 2000 to 2018 (Takatani et al. 2014; Iida et al. 2015, 2021). For the BioCO_2_, we used averaged monthly flux maps based on the inversion for the period of 2006–2008, conducted with NICAM-TM (Niwa et al. 2012). As for the global surface CH_4_ fluxes, we also used monthly inversion flux maps computed by the NICAM-TM 4D-Var system with a set of global surface observations including HAT and YON for the period from 2000 to 2017 (Niwa et al. 2017a, b; Saunois et al. 2020). We did not use the inversion BioCO_2_ fluxes in this study because the objective of the simulation was only to investigate the relationship between the ΔCO_2_/ΔCH_4_ ratio at the observation sites and the CO_2_/CH_4_ emission ratio in China. Thus, the CO_2_ and CH_4_ fluxes did not need to exactly represent the actual temporal variations during the analysis period. Only the seasonal cycles of those CO_2_ and CH_4_ fluxes were made consistent with the observations by using the inversion results of Niwa et al. (2012) and Saunois et al. (2020), respectively.

In this study, we simulated the atmospheric CO_2_ and CH_4_ mole fractions at HAT for 2000–2021 by using the corresponding climate dataset from the JRA-55 reanalysis and the above CO_2_ and CH_4_ flux data. When there were no flux data for the corresponding years, we repeatedly used the latest flux maps instead: FFCO_2_ emission and ocean CO_2_ flux maps from 2018, and CH_4_ flux maps from 2017. The temporal changes in the monthly fluxes of the FFCO_2_, land BioCO_2_, and CH_4_ from China used in the simulation are plotted in Additional file 1: Fig. S1. This set of temporally increasing FFCO_2_ emissions is hereinafter referred to as Set A. It should be noted that we do not necessarily need prior information on emissions for the target years 2020, 2021, and 2022 in this study, because only relative emission changes are estimated based on the simulated relationship between the emission ratios and the variability ratios.

The time series of the simulated atmospheric CO_2_ and CH_4_ mole fractions at HAT during JFM in 2020 and 2021 are plotted in Fig. 2. The simulations generally well reproduced the observed synoptic-scale variations of both CO_2_ and CH_4_. Using these simulated time series based on the FFCO_2_ emissions of Set A, we examined the relationship between the ΔCO_2_/ΔCH_4_ ratio and the CO_2_/CH_4_ emission ratio in China. Additionally, we simulated the atmospheric CO_2_ and CH_4_ at HAT in January, February, and March 2020 and 2021 by changing the FFCO_2_ emissions from China in 2018 to 55, 70, 85, 115, and 130% emissions and examined the relationship between the simulated variability ratio and emission ratio using these modified FFCO_2_ emissions. This set of modified FFCO_2_ emissions is hereinafter referred to as Set B. Note that we used the emissions from the whole mainland of China to calculate the emission ratio and we multiplied the emissions from the entire China by single factors to prepare the modified FFCO_2_ emissions. The simulated relationship between the variability ratio and the emission ratio can also be applied to the observations at YON because the simulated ΔCO_2_/ΔCH_4_ ratios at HAT and YON were almost the same, reflecting the relatively short distance between HAT and YON (about 90 km) compared to the geographical relation between both sites and continental China. The observed data for both sites show almost identical synoptic-scale variations except for diurnal cycles.

## Results and discussion

### Temporal change in the ΔCO_2_/ΔCH_4_ ratios at HAT and YON

**Fig. 3:**
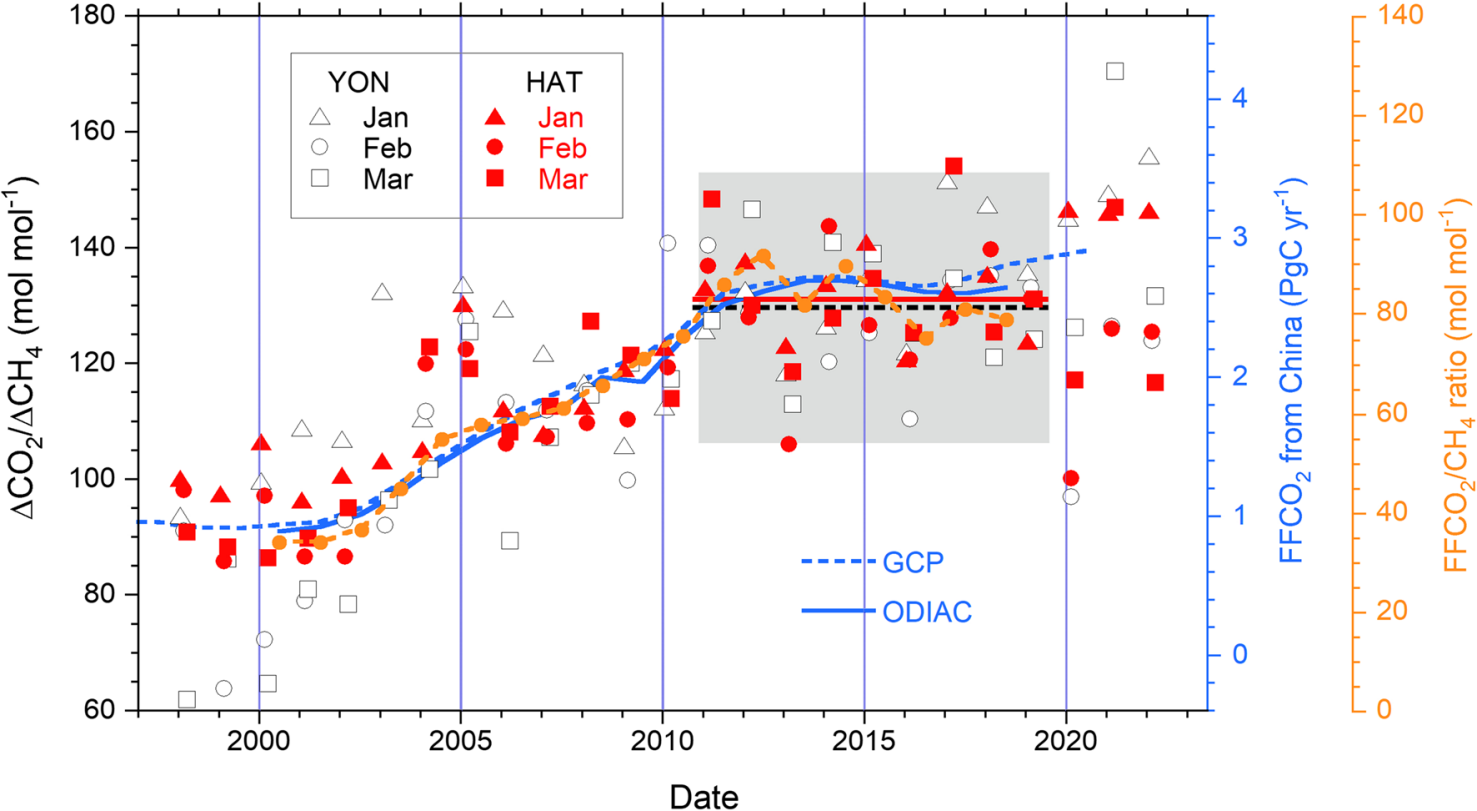
Temporal changes in the monthly ΔCO_2_/ΔCH_4_ ratios based on the observations at HAT and YON. The monthly averages at HAT (closed red symbols) and at YON (open black symbols) for January (triangles), February (circles), and March (squares) from 1998 to 2022 are plotted. Solid red and dashed black lines represent the average values of the monthly ΔCO_2_/ΔCH_4_ ratios during a 9-year period (2011–2019) for HAT and YON, respectively. The gray-shaded area represents the 95% confidence interval for the average for YON. The dashed and solid blue lines represent the FFCO_2_ emissions from China (right blue Y-axis) taken from GCP (Friedlingstein, et al. 2022) and ODIAC (Oda et al. 2018), respectively. The orange circles connected with the dashed orange line represent the temporal change in the FFCO_2_/CH_4_ emission ratios, which were averages based on the emissions from China used in the model simulation in this study (Additional file 1: Fig. S1) for the 3 months

The monthly mean values of the ΔCO_2_/ΔCH_4_ ratio observed at HAT and YON between 1998 and 2022 are plotted in Fig. 3. As our previous study (Tohjima et al. 2022) suggested, there are considerable similarities in the temporal change between HAT and YON; the ΔCO_2_/ΔCH_4_ ratios at both sites show a gradual increase in the 2000s and rather stable values after 2011. These trends in the variability ratio are mostly attributed to the changes in the FFCO_2_ emissions from China (Tohjima et al. 2014, 2020). In fact, the pattern of the temporal changes in the annual FFCO_2_ emissions from China taken from estimations of the Global Carbon Project (GCP) (Friedlingstein et al. 2022) and ODIAC (Oda et al. 2018) generally agrees with that of the ΔCO_2_/ΔCH_4_ ratio (Fig. 3). In Fig. 3, we also plotted the average ratios of the estimated FFCO_2_ and CH_4_ emissions from China for JFM between 2000 and 2018. Note that these emission ratios were based on the emission estimates of Set A used in the model simulation of this study (Additional file 1: Fig. S1). The estimated CH_4_ emissions have a gradually increasing trend (Additional file 1: Fig. S1), but the FFCO_2_/CH_4_ emission ratio for China shows a similar temporal pattern to the FFCO_2_ emissions from China (Fig. 3), indicating that the trend in emission ratio is dominated by the trend in FFCO_2_ emissions.

Previous studies also showed marked decreases in the ΔCO_2_/ΔCH_4_ ratios at HAT and YON in February 2020, when the COVID-19-related nationwide lockdown in China considerably reduced the FFCO_2_ emissions (Tohjima et al. 2020; 2022). In Fig. 3, the averages of the ΔCO_2_/ΔCH_4_ ratios for HAT and YON during the preceding 9-year period (2011–2019) are drawn with a 95% confidence interval for YON. The ΔCO_2_/ΔCH_4_ ratios at both sites fall below the 95% confidence limit in February 2020. In contrast, the monthly mean ΔCO_2_/ΔCH_4_ ratios at HAT and YON during JFM in 2021 and 2022 returned to the previous 9-year (2011–2019) level or higher. This suggests that the FFCO_2_ emissions from China in early 2021 returned to the same level as or higher than before the COVID-19 lockdown.

**Fig. 4:**
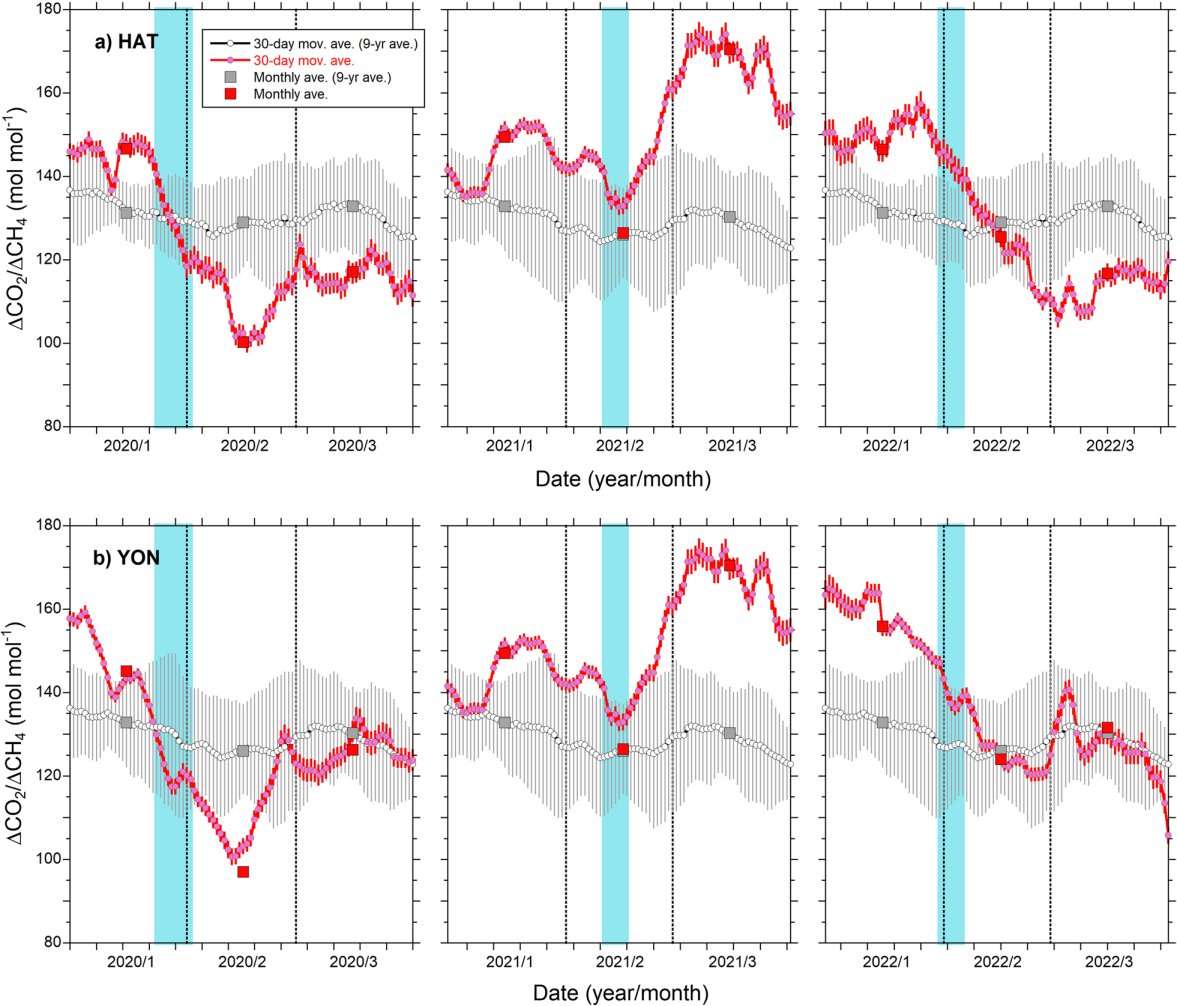
Consecutive changes in the ΔCO_2_/ΔCH_4_ ratio during January–March 2020, 2021, and 2022. The changes based on the observations at (**a**) HAT and (**b**) YON are depicted. The pink dots with lines and the red squares represent the 30-day moving averages and the monthly averages of the ΔCO_2_/ΔCH_4_ ratio, respectively. The gray open circles are the averages of the 30-day moving averages of the ΔCO_2_/ΔCH_4_ ratio for the preceding 9 years (2011–2019) and the vertical bars are their uncertainties. The temporal resolution of the consecutive changes is ± 15 days. The light-blue-shaded areas correspond to the Chinese New Year holidays

To take a closer look at the temporal changes in the ΔCO_2_/ΔCH_4_ ratio during JFM in 2020, 2021, and 2022, the 30-day moving averages of the ΔCO_2_/ΔCH_4_ ratios for HAT and YON are depicted in Fig. 4. For comparison, averages of the ΔCO_2_/ΔCH_4_ ratios for the preceding 9 years (2011–2019) are also drawn together with the standard deviations in the figure. The ΔCO_2_/ΔCH_4_ ratios show decreases between January and February 2020, minima in the middle of February, and gradual increases toward the preceding 9-year averages in March 2020. Previous studies (Tohjima et al. 2020, 2022) suggested that the above temporal patterns of the consecutive ΔCO_2_/ΔCH_4_ ratios were consistent with the estimated change in the FFCO_2_ emissions from China based on a study by Le Quéré et al. (2020). On the other hand, the consecutive ΔCO_2_/ΔCH_4_ ratios during JFM in 2021 are larger than the preceding 9-year average and spread. The local minimum in the middle of February 2021 might be related to the reduction in economic activity during the Chinese New Year holidays, which were from February 11 to 17, 2021 (see Fig. 4). The period of the Chinese New Year holidays was from January 24 to February 2 in 2020, of which the last 3 days were extended holidays to fight the spread of COVID-19. These results seem to be consistent with the recovery of economic activity in China from the influence of the COVID-19 pandemic in early 2021.

It is noteworthy that the consecutive ΔCO_2_/ΔCH_4_ ratios in 2022, which were higher than the preceding 9-year average in January and decreased in February, reached the preceding 9-year level or lower in March. Since the COVID-19 infection spread again mostly in Shanghai after March, the reduced ΔCO_2_/ΔCH_4_ ratios might reflect the decrease in FFCO_2_ emissions associated with the confinements of the socioeconomic activities in China (Myllyvirta 2022).

### Simulated ΔCO_2_/ΔCH_4_ ratio

**Fig. 5:**
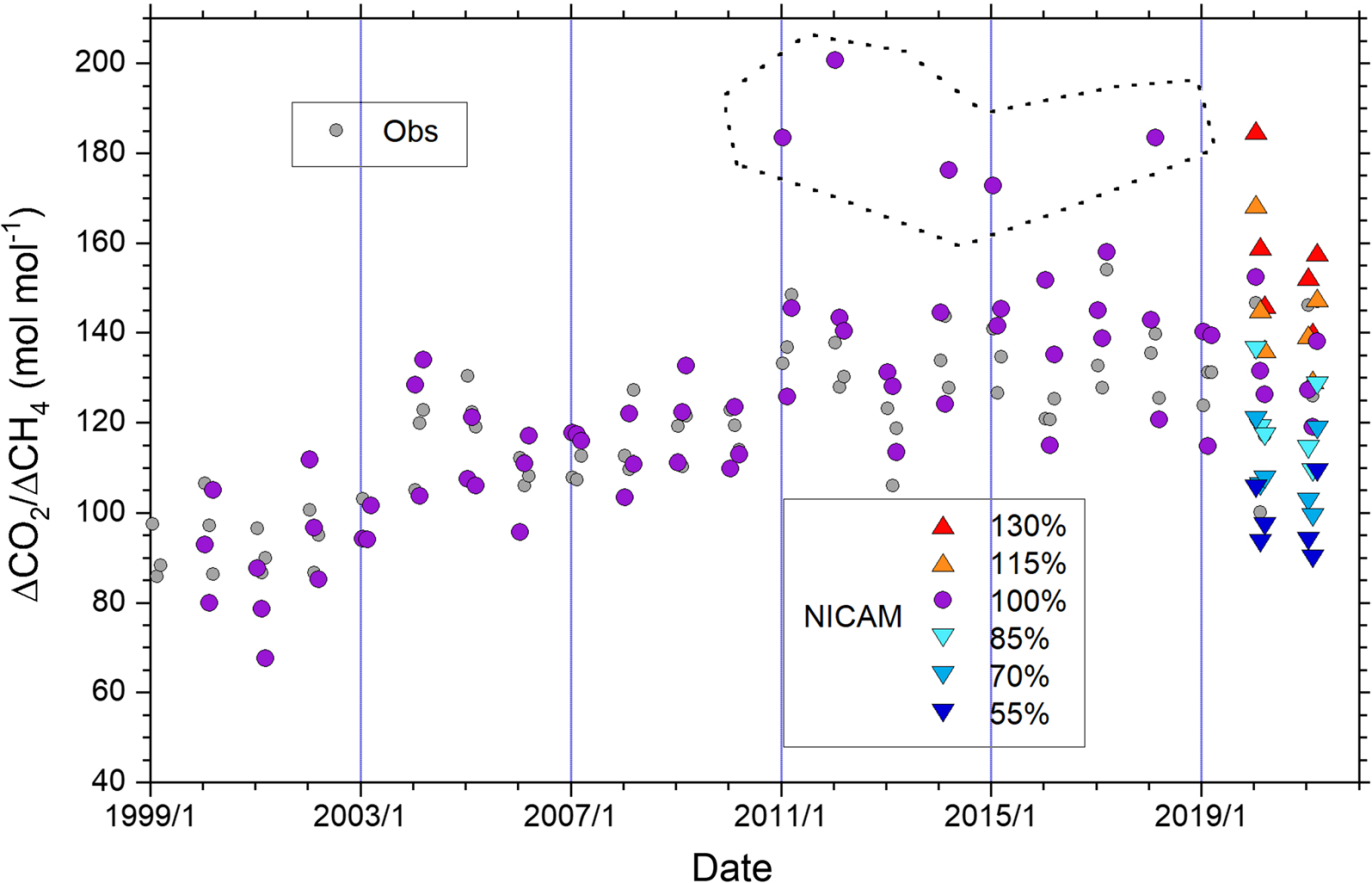
Temporal change in the monthly ΔCO_2_/ΔCH_4_ ratios during January–March from 2000 to 2021. The simulated monthly ΔCO_2_/ΔCH_4_ ratios based on the FFCO_2_ emissions of Set A are plotted as purple circles. The observed monthly ΔCO_2_/ΔCH_4_ ratios at HAT are plotted as gray circles for comparison. The purple circles surrounded by the dotted line represent the outliers, which are more than 20 mol mol^−1^ larger than the corresponding observed ratios. The color-coded triangles represent the simulated results based on the FFCO_2_ emissions of Set B from 55 to 130% for the meteorological fields of 2020 and 2021

Using the simulated atmospheric CO_2_ and CH_4_ at HAT for the period from 2000 to 2021 based on the FFCO_2_ emissions of Set A, we calculated the monthly averages of the ΔCO_2_/ΔCH_4_ ratio from January to March in the same way as the observed data at HAT were computed. The simulated and observed monthly averaged ΔCO_2_/ΔCH_4_ ratios during 2000–2021 are shown in Fig. 5. The simulated ΔCO_2_/ΔCH_4_ ratios roughly trace the observed increasing trend in the 2000s and plateau after 2011 except for five points enclosed by dotted lines, which are more than 20 mol mol^−1^ larger than the corresponding observed ratios. These discrepancies were caused by the fact that the monthly ΔCO_2_/ΔCH_4_ ratios included some extraordinarily large values with very small CH_4_ variability. These erroneous ΔCO_2_/ΔCH_4_ ratios might be attributed to uncertainties in model transport or flux distributions used in the simulation or both. Thus, we rejected these five data as outliers in the subsequent analysis. The observed and simulated ΔCO_2_/ΔCH_4_ ratios without the above-mentioned five outliers show a clear positive correlation with a correlation coefficient of 0.74 and a slope of 1.2 ± 0.1 (Additional file 1: Fig. S2). The regression slope was determined by the RMA method, and the uncertainties (1*σ*) of the parameters were evaluated by a bootstrap method, in which the regression calculations were repeatedly applied to the datasets prepared by iterative resampling with replacement (*n* = 10,000). We used the above approach also for the regression analyses in the following section. Additional sensitivity simulations are also shown for the ΔCO_2_/ΔCH_4_ ratio based on the FFCO_2_ emissions of Set B and the meteorological reanalysis data during 2020–2021 in Fig. 5.

**Fig. 6:**
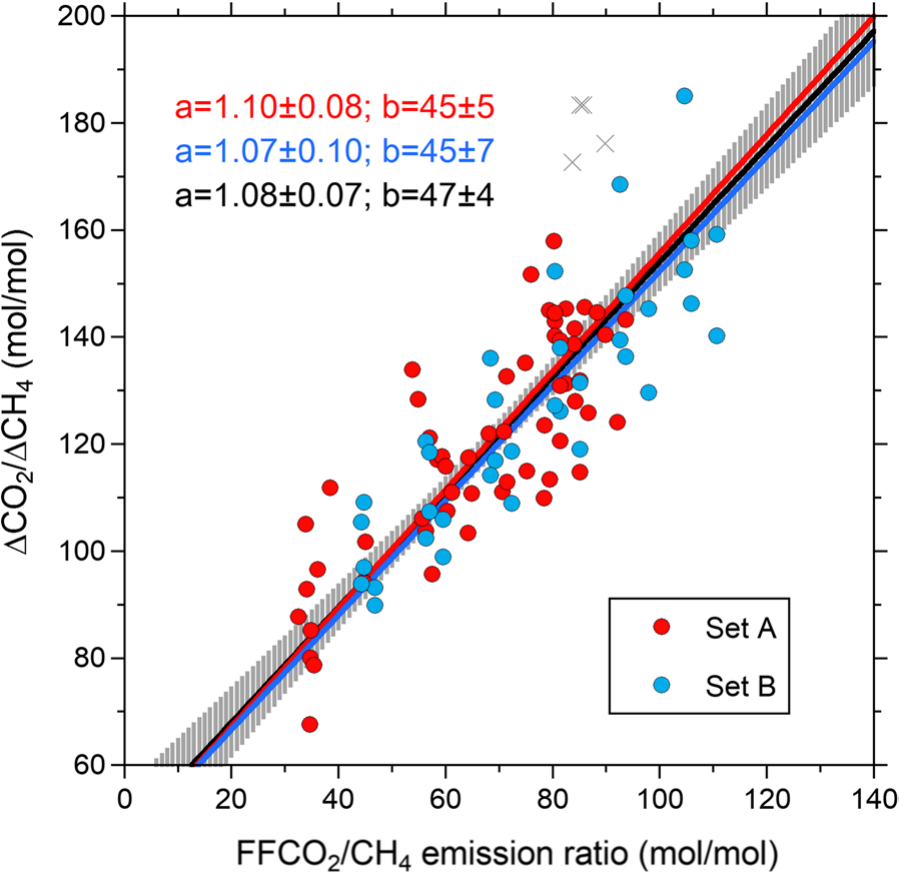
Scatter plot between the FFCO_2_/CH_4_ emission ratios in China and the simulated ΔCO_2_/ΔCH_4_ ratios for HAT. The plots based on the FFCO_2_ emissions of Set A and Set B are shown as red and blue circles, respectively. The red, blue, and black lines represent linear regression lines for the plots based on Set A, Set B, and their combined set, respectively. The values for “a” and “b” in the figure represent the related slopes and y-axis intercepts, respectively. The gray vertical bars are estimated uncertainties (1*σ*) for the regression line based on the total FFCO_2_ data. Gray crosses are the outliers

The relationship between the simulated ΔCO_2_/ΔCH_4_ ratios and the FFCO_2_/CH_4_ emission ratios in China is shown in Fig. 6, where the simulated results for 2000–2021 and 2020–2021 based on the FFCO_2_ emissions of Set A and Set B, respectively, are plotted. As expected, both datasets show positive and consistent correlations and slopes. From the linear regression analysis, we obtained slopes and y-intercepts of 1.10 ± 0.08 and 45 ± 5 (mol mol^−1^), respectively, for Set A and those of 1.07 ± 0.10 and 45 ± 7 (mol mol^−1^), respectively, for Set B. There are no statistically significant differences between the two regression lines (*p* = 0.63), suggesting that the contribution of the year-to-year differences in atmospheric transport does not strongly influence the relationship between the ΔCO_2_/ΔCH_4_ ratios and the FFCO_2_/CH_4_ emission ratios. Such characteristics are brought about by the very fact that calculating the variability ratio cancels out the transport influences. Therefore, by combining Set A and Set B for the simulated relationship between the ΔCO_2_/ΔCH_4_ ratio and FFCO_2_/CH_4_ emission ratio, we obtained a single regression line with a slope of 1.08 ± 0.07 and a y-intercept of 47 ± 4 (mol mol^−1^) (Fig. 6). The results of the regression analysis are summarized in Table 1.

**Table 1:** Summary of the regression analysis of the simulated ΔCO_2_/ΔCH_4_ ratio at HAT using three sets of FFCO_2_ emissions (A, B, and A + B) against three sets of emission ratios, FFCO_2_/CH_4_, (FFCO_2_ + BioCO_2_)/CH_4_, and FFCO_2_ in China

Emission ratio or emission	Set A (2000–2021)		Set B (2020–2021)		Set A + B		*p* value^c^
	Slope	Intercept^a^	Slope	Intercept^a^	Slope	Intercept^a^	
FFCO_2_/CH_4_	1.10 ± 0.08	45 ± 5	1.07 ± 0.10	45 ± 7	1.08 ± 0.07	47 ± 4	0.63
(FFCO_2_ + BioCO_2_)/CH_4_	1.19 ± 0.08	16 ± 7	1.07 ± 0.10	26 ± 9	1.12 ± 0.07	22 ± 6	0.51
FFCO_2_	0.36 ± 0.02^b^	58 ± 4	0.40 ± 0.04^b^	46 ± 7	0.37 ± 0.02^b^	56 ± 04	0.04

^a^Units of intercepts are given in mol mol^−1^

^b^Units of slopes for FFCO_2_ emission are given in (mol mol^−1^)/TgC

^c^p values are for the hypothesis that there is no significant difference between the slopes for the time-dependent fluxes and modified fluxes

The simulated ΔCO_2_/ΔCH_4_ ratios also showed linear relationships to the (FFCO_2_ + BioCO_2_)/CH_4_ emission ratios and FFCO_2_ emissions in China, as shown in Additional file 1: Fig. S3. These results are summarized in Table 1. The difference of the y-intercepts for the FFCO_2_/CH_4_ from those for the (FFCO_2_ + BioCO_2_)/CH_4_ corresponds to the influence of the BioCO_2_ fluxes on the ΔCO_2_/ΔCH_4_ ratios. The regression slopes for the emission ratios are close to unity, and the y-intercepts for the (FFCO_2_ + BioCO_2_)/CH_4_ are roughly close to the origin, indicating that the ΔCO_2_/ΔCH_4_ ratios at HAT straightforwardly reflect the CO_2_/CH_4_ emission ratios in China, as was indicated in a previous study (Tohjima et al. 2014). As for the relationship to the FFCO_2_ emissions, there is a significant difference (*p* < 0.05) between the regression lines for the ΔCO_2_/ΔCH_4_ ratios derived from the FFCO_2_ emissions of Set A and Set B, indicating that the different rate of increase in the CH_4_ emissions and potentially the spatial heterogeneity in the change rates of the realistic FFCO_2_ emissions of Set A contribute to the temporal change in the ΔCO_2_/ΔCH_4_ ratios. In the following sections, assuming that the land biospheric CO_2_ fluxes from China have no interannual variations, we used the linear relationship between the ΔCO_2_/ΔCH_4_ ratios and the FFCO_2_/CH_4_ emission ratio to evaluate the change in the FFCO_2_/CH_4_ emission ratio in China.

Although the JFM chosen for the analysis corresponds to the period when the biotic activities are relatively dormant, there can be a measurable interannual variability in the BioCO_2_ and CH_4_ fluxes (Additional file 1: Fig. S1). From the inversely estimated BioCO_2_ and CH_4_ fluxes from China after 2011 based on NICAM-TM (see Additional file 1: Fig. S1), we obtained averages and standard deviations (1*σ*) of 2.0 ± 1.3 TgC day^−1^ and 0.118 ± 0.008 TgCH_4_ day^−1^ for the BioCO_2_ and CH_4_ fluxes, respectively. These standard deviations for the BioCO_2_ and CH_4_ fluxes correspond to about ± 14% and ± 7% variations in the (FFCO_2_ + BioCO_2_)/CH_4_ emission ratio in China in the recent decade, respectively. Meanwhile, the CH_4_ emissions from China for the 3 months are mostly derived from anthropogenic sources, including coal mining, landfills, enteric fermentation, and other anthropogenic sources, except for paddy fields (e.g., Ito et al. 2019). Among these anthropogenic sources, coal mining is the largest source in China, contributing about 40% of the total emission during JFM. Since the increase in coal consumption historically enhanced the FFCO_2_ and CH_4_ emissions in China, it was inferred that these emissions positively correlated, as was pointed out by Saeki and Patra (2017). Therefore, such a positive correlation of the emissions might, to some extent, attenuate the temporal change in the observed ΔCO_2_/ΔCH_4_ ratios at HAT and YON. The influence of the correlative change in the CH_4_ emissions on the ΔCO_2_/ΔCH_4_ ratios in this model simulation can be evaluated from the linear relationships of the ΔCO_2_/ΔCH_4_ ratios against the FFCO_2_ emissions of Set A and Set B listed in Table 1. For the same change in the FFCO_2_ emissions from 220 TgC, the change in the simulated ΔCO_2_/ΔCH_4_ ratios for Set A is about 8% lower than that for Set B.

### Estimation of the FFCO_2_/CH_4_ emission ratio in China

#### Conversion of the ΔCO_2_/ΔCH_4_ ratio to FFCO_2_/CH_4_ emission ratio

Using the observed monthly averages of the ΔCO_2_/ΔCH_4_ ratios at HAT and YON for JFM in 2020, 2021, and 2022 and the preceding 9-year (2011–2019) averages for JFM, we evaluated the changes in the FFCO_2_/CH_4_ emission ratio in China in 2020, 2021, and 2022 in comparison with the preceding 9-year (2011–2019) averages (Table 2). Here, we adopted the standard errors of the monthly averages of the selected regression slopes (Sect. 2.2) and the standard deviations for the 9-year averages of the monthly ΔCO_2_/ΔCH_4_ ratios as the respective uncertainties. These ΔCO_2_/ΔCH_4_ ratios were translated into the FFCO_2_/CH_4_ emission ratio in China by the linear function deduced in the previous section. Then, we calculated the change rate of the FFCO_2_/CH_4_ emission ratios compared to the 9-year averages at HAT and YON and their weighted averages. Here we set the reciprocal of the square of the uncertainty associated with each FFCO_2_/CH_4_ emission ratio for the weight. Table 2 lists these estimated changes in the FFCO_2_/CH_4_ emission ratio ranging from − 36 to 48%.

**Table 2:** Observed ΔCO_2_/ΔCH_4_ ratio and estimated change in the FFCO_2_/CH_4_ emission ratio in China from the preceding 9-year average

Date (year/month)	Monthly ΔCO_2_/ΔCH_4_ (mol mol^−1^)		Estimated change in the FFCO_2_/CH_4_ emission ratio from the preceding 9 years (%)		
	HAT	YON	HAT	YON	Weighted Ave
2020/01	147 ± 2	145 ± 1	18 ± 10	14 ± 15	17 ± 8
2020/02	100 ± 2	97 ± 2	− 35 ± 9	− 36 ± 10	− 36 ± 7
2020/03	117 ± 2	126 ± 2	− 18 ± 11	− 5 ± 13	− 12 ± 8
2021/01	146 ± 2	149 ± 1	18 ± 10	19 ± 15	18 ± 8
2021/02	126 ± 2	126 ± 1	− 4 ± 13	1 ± 15	− 2 ± 10
2021/03	147 ± 2	170 ± 2	16 ± 16	48 ± 20	29 ± 12
2022/01	146 ± 2	156 ± 1	18 ± 10	27 ± 16	20 ± 9
2022/02	126 ± 3	124 ± 1	− 4 ± 13	− 2 ± 14	− 3 ± 10
2022/03	117 ± 2	132 ± 2	− 19 ± 11	2 ± 13	− 10 ± 9
2011–2019/01	131 ± 7	133 ± 11	–	–	–
2011–2019/02	129 ± 11	126 ± 12	–	–	–
2011–2019/03	133 ± 11	130 ± 11	–	–	–

In our previous study (Tohjima et al. 2020), using the same monthly average ΔCO_2_/ΔCH_4_ ratios at HAT and an atmospheric model simulation, we estimated the relative changes in the FFCO_2_ emissions from China to be − 32 ± 12% for February and − 19 ± 15% for March 2020 under the assumption of invariable CH_4_ emissions. The slight differences in the FFCO_2_ emission changes in this study are attributed to the different approach of the previous study, in which the FFCO_2_ emissions from China were reduced in proportion to the bottom-up estimate based on the economic activity data of Le Quéré et al. (2020

**Fig. 7:**
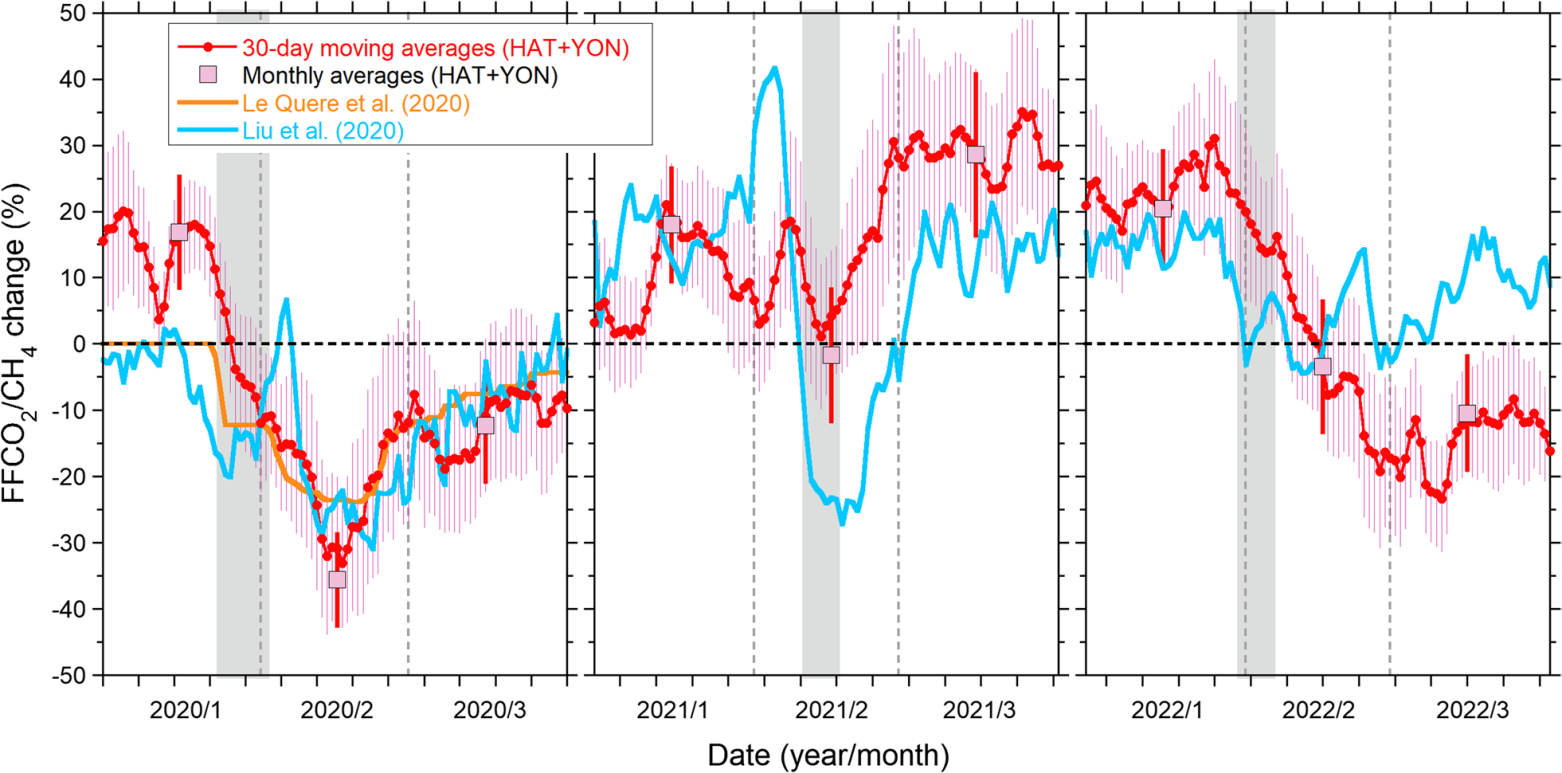
Estimated FCO_2_/CH_4_ emission change in China based on the ΔCO_2_/ΔCH_4_ ratios observed at HAT and YON. The estimated results for 3 months (January, February, and March) in 2020, 2021, and 2022 are depicted in the left, middle, and right panels, respectively. The red circles with red lines and pink squares represent the estimates based on the 30-day moving averages and monthly averages, respectively, of the observed ΔCO_2_/ΔCH_4_ ratios. The vertical bars represent the uncertainties. For comparison, the temporal changes in FFCO_2_ emissions from China based on the bottom-up estimates of Le Quéré et al. (2020) (orange line) and Liu et al. (2020) (light blue line) are also shown. The gray-shaded areas correspond to the Chinese New Year holidays

We also estimated the consecutive changes in the FFCO_2_/CH_4_ emission ratio based on the 30-day moving averages of the ΔCO_2_/ΔCH_4_ ratio at HAT and YON for the JFM in 2020, 2021, and 2022. As was done for the monthly averages, we converted the consecutive variability ratios to emission ratios and computed the rate of change in the emission ratios for the preceding 9-year averages. The estimated rates of change in the FFCO_2_/CH_4_ emission ratio for HAT and YON are depicted in Additional file 1: Fig. S4, and their weighted averages with the propagated uncertainties (1*σ*) are shown in Fig. 7. Hereinafter, we discuss the weighted averages of the estimated FFCO_2_/CH_4_ emission ratios, which can be interpreted as the change in the FFCO_2_ emissions in China under the assumption of no interannual CH_4_ emission change during JFM.

#### FFCO_2_/CH_4_ emission change in 2020

The estimated monthly change in the FFCO_2_/CH_4_ emission ratios for 2020 compared with the preceding 9-year averages was 17 ± 8% for January, − 36 ± 7% for February, and − 12 ± 8% for March (Table 2). The average change for the 3 months is − 10 ± 9% compared with the preceding 9-year average. The value is consistent with previous estimates based on bottom-up approaches: − 10.1% (− 4.6 to − 16.5%) by Le Quéré et al. (2020) and − 13% by Liu et al. (2020) for JFM. Note that these bottom-up values were reported as the changes in emissions from the previous year (2019).

As was discussed in Tohjima et al. (2020), the marked decrease in February 2020 corresponded to the period of the nationwide lockdown in China, and the slight recovery in March corresponded to the transition period to normal conditions. Such a temporal change is more clearly shown in the plot of the consecutive estimation of the FFCO_2_/CH_4_ emission ratio (Fig. 7). For comparison, the temporal changes in the FFCO_2_ emissions from China based on bottom-up estimates (Le Quéré et al. 2020; Liu et al. 2020) are also depicted in the figure. Both bottom-up estimates begin to decrease in late January, reach a minimum in the middle of February, then gradually return to the previous emission level (0%), although the estimate of Liu et al. (2020) shows a sharp maximum at the beginning of February. The sharp maximum corresponds to the estimated FFCO_2_ emission minimum of the previous year (2019), which was attributed by Liu et al. (2020) to the reduction in economic activity in China during the Chinese New Year holidays from February 4 to 10, 2019. Except for the sharp maximum, our estimation agrees well with the bottom-up estimations. These results seem to support the reliability of our simple estimation approach based on the atmospheric ΔCO_2_/ΔCH_4_ ratio.

#### FFCO_2_/CH_4_ emission change in 2021

The estimated FFCO_2_/CH_4_ emission ratios for 2021 were equal to or larger than the preceding 9-year average; the average change was 18 ± 8% for January, − 2 ± 10% for February, and 29 ± 12% for March, and the average change for JFM is 15 ± 10%. The relatively lower ratio for February than those for January and March may be attributed to the temporal decrease in FFCO_2_ emissions during the Chinese New Year holidays. From the extended estimates of the bottom-up study of Liu et al. (2020) (https://www.carbonmonitor.org.cn), we obtained a monthly change of 16% for January, − 1% for February, and 14% for March relative to 2019, and the average change for JFM was 10%, which is again consistent with the estimations of this study. The consecutive variations in 2021 are shown in Fig. 7. Compared with the bottom-up estimate of Liu et al. (2020), the temporal variability of our estimation is rather suppressed, especially during the period related to the Chinese New Year holidays. The difference is partially explained by the low temporal resolution (± 15 days) of our estimation. In addition, our estimated FFCO_2_ emissions for March 2021 are slightly larger than the bottom-up estimates of Liu et al. (2020). These enhanced estimates were supported by the ΔCO_2_/ΔCH_4_ ratios observed at both HAT and YON (see Additional file 1: Fig. S4). Note that the ΔCO_2_/ΔCH_4_ ratios at YON were more than double those at HAT. Possibly, influences from local emissions were not sufficiently eliminated (see Sect. 2.2), enhancing the ΔCO_2_/ΔCH_4_ ratios at YON in March 2021. Our observational result, although still having a large uncertainty, suggests that the FFCO_2_ emissions from China considerably rebounded in early 2021 despite the global effort to reduce GHGs emissions.

#### FFCO_2_/CH_4_ emission change in 2022

The estimated changes in the monthly FFCO_2_/CH_4_ emission ratios for 2022 were 20 ± 9%, − 3 ± 10%, and − 10 ± 9% for January, February, and March, respectively, and the average change for JFM is 2 ± 9%. The monthly FFCO_2_ emission changes relative to 2019 taken from the extended estimates of the bottom-up study of Liu et al. (2020) were 15% for January, 3% for February, and 8% for March, and the average change for JFM was 9%, which are again consistent with the estimations of this study except for March. The consecutive variations in our emission estimate for 2022 shown in Fig. 7 persistently decrease in February even after the Chinese New Year holidays and maintain a low level in March, whereas the bottom-up result of Liu et al. (2020) shows a gradual increase after the Chinese New Year holidays. The emissions from Shanghai strongly affected the ΔCO_2_/ΔCH_4_ ratios observed at HAT and YON because of the relatively short distance. Therefore, our estimated changes based on atmospheric observations might reflect the FFCO_2_ emission decreases due to the spread of COVID-19 in Shanghai after March.

## Conclusions

We developed a near-real-time estimation method for the change in the FFCO_2_ emissions from China based on the synoptic-scale variability ratio of atmospheric CO_2_ and CH_4_ (ΔCO_2_/ΔCH_4_ ratio) in January, February, and March (JFM) on two remote islands in Japan, HAT and YON. From simulation results based on an atmospheric transport model (NICAM-TM) with all components of realistic CO_2_ and CH_4_ fluxes, we found a linear relationship between the monthly averaged ΔCO_2_/ΔCH_4_ ratios and the FFCO_2_/CH_4_ emission ratios in China. We used this simulated linear relationship to translate the observed ΔCO_2_/ΔCH_4_ ratio into FFCO_2_/CH_4_ emission ratios under the assumption of no interannual BioCO_2_ change during JFM. The change in the estimated FFCO_2_/CH_4_ emission ratio can be interpreted as the change in the FFCO_2_ emissions by assuming no interannual CH_4_ emission change during JFM. Because this method is simple compared to the inverse method, near-real-time monitoring is feasible.

Using the developed method, we estimated the change in the FFCO_2_/CH_4_ emission ratios for 2020, 2021, and 2022 with respect to the average emission ratios for the preceding 9-year period (2011–2019), during which relatively stable ΔCO_2_/ΔCH_4_ ratios were observed at both HAT and YON. The resulting changes in the FFCO_2_ emissions for January, February, and March were 17 ± 8%, − 36 ± 7%, and − 12 ± 8%, respectively, in 2020 (− 10 ± 9% for JFM overall), 18 ± 8%, − 2 ± 10%, and 29 ± 12%, respectively, in 2021 (15 ± 10% for JFM overall), and 20 ± 9%, − 3 ± 10%, and − 10 ± 9%, respectively, in 2022 (2 ± 9% for JFM overall). The estimations for 2020 of not only the average change but also the temporal pattern of the FFCO_2_ emission change agreed well with the reported estimations based on bottom-up studies (Le Quéré et al. 2020 and Liu et al. 2020). Therefore, our estimations for 2021 strongly suggest that FFCO_2_ emissions from China rebounded with the recovery of the socioeconomic activities after the COVID lockdown in China. However, our estimated FFCO_2_ change showed a slight decrease in March 2022, suggesting that the FFCO_2_ emissions from China were still affected by the infection status of COVID-19 in China.

This early estimation method proposed in this study only gives us quick but rough estimations because of a variety of assumptions. Particularly, the assumption that BioCO_2_ and CH_4_ have no interannual variations should be validated in future studies to refine the estimated FFCO_2_ change based on more comprehensive analyses. Additionally, we could probably overcome the limitation of the short estimation period of this study by analyzing the atmospheric observations from tall towers and aircraft with reduced interferences from the local biosphere and with large footprint areas in continental China. Nevertheless, we consider this estimation method useful for the verification of the GHG emission mitigation strategy in China or elsewhere with strategically positioned measurement sites.

## Supplementary Information

**Additional file 1**. **Fig. S1.** Temporal changes in the monthly emissions of (a) FFCO_2_, (b) BioCO_2_, and (c) CH_4_ from China used in the simulation of this study. **Fig. S2.** Scatter plot of the relationship of the ΔCO_2_/ΔCH_4_ ratios (red circles) between the observation and simulation. **Fig. S3.** Scatter plots of the simulated ΔCO_2_/ΔCH_4_ ratios for HAT to (a) the (FFCO_2_+BioCO_2_)/CH_4_ emission ratios and (b) the FFCO2 emissions in China. **Fig. S4.** Temporal changes of the FFCO_2_/CH_4_ emission ratio relative to the preceding 9-year (2011-2019) averages during the three-month period (January-March) in 2020, 2021, and 2022 for (a) HAT and (b) YON.

## Data Availability

The time series of the atmospheric CO_2_ and CH_4_ mole fractions at HAT are available through the NIES database, Global Environmental Database (GED) (https://db.cger.nies.go.jp/ged/en/index.html). The time series of the atmospheric CO_2_ and CH_4_ mole fractions at YON are available through the Web site of the World Data Centre for Greenhouse Gases (WDCGG). (https://xml.kishou.go.jp/).

## Abbreviations

HAT: Hateruma Island
YON: Yonaguni Island
NIES: National Institute for Environmental Studies
JMA: Japan Meteorological Agency
RMA: Reduced major axis regression
NICAM-TM: Nonhydrostatic ICosahedral Atmospheric Model (NICAM)-based transport model
ODIAC: Open-source Data Inventory for Anthropogenic CO_2_
