# Supplementary material for: Near-real-time estimation of fossil fuel CO2 emissions from China based on atmospheric observations on Hateruma and Yonaguni Islands, Japan
Source: Prog Earth Planet Sci. 2023 Mar 2;10(1):10. doi: 10.1186/s40645-023-00542-6 (PMC9978285; doi:10.1186/s40645-023-00542-6)
Supplement: Supplementary file 1 — Additional file 1. Fig. S1. Temporal changes in the monthly emissions of (a) FFCO2, (b) BioCO2, and (c) CH4 from China used in the simulation of this study. Fig. S2. Scatter plot of the relationship of the ΔCO2/ΔCH4 ratios (red circles) between the observation and simulation. Fig. S3. Scatter plots of the simulated ΔCO2/ΔCH4 ratios for HAT to (a) the (FFCO2+BioCO2)/CH4 emission ratios and (b) the FFCO2 emissions in China. Fig. S4. Temporal changes of the FFCO2/CH4 emission ratio relative to the preceding 9-year (2011-2019) averages during the three-month period (January-March) in 2020, 2021, and 2022 for (a) HAT and (b) YON. [file 40645_2023_542_MOESM1_ESM.docx]

Supplementary figures


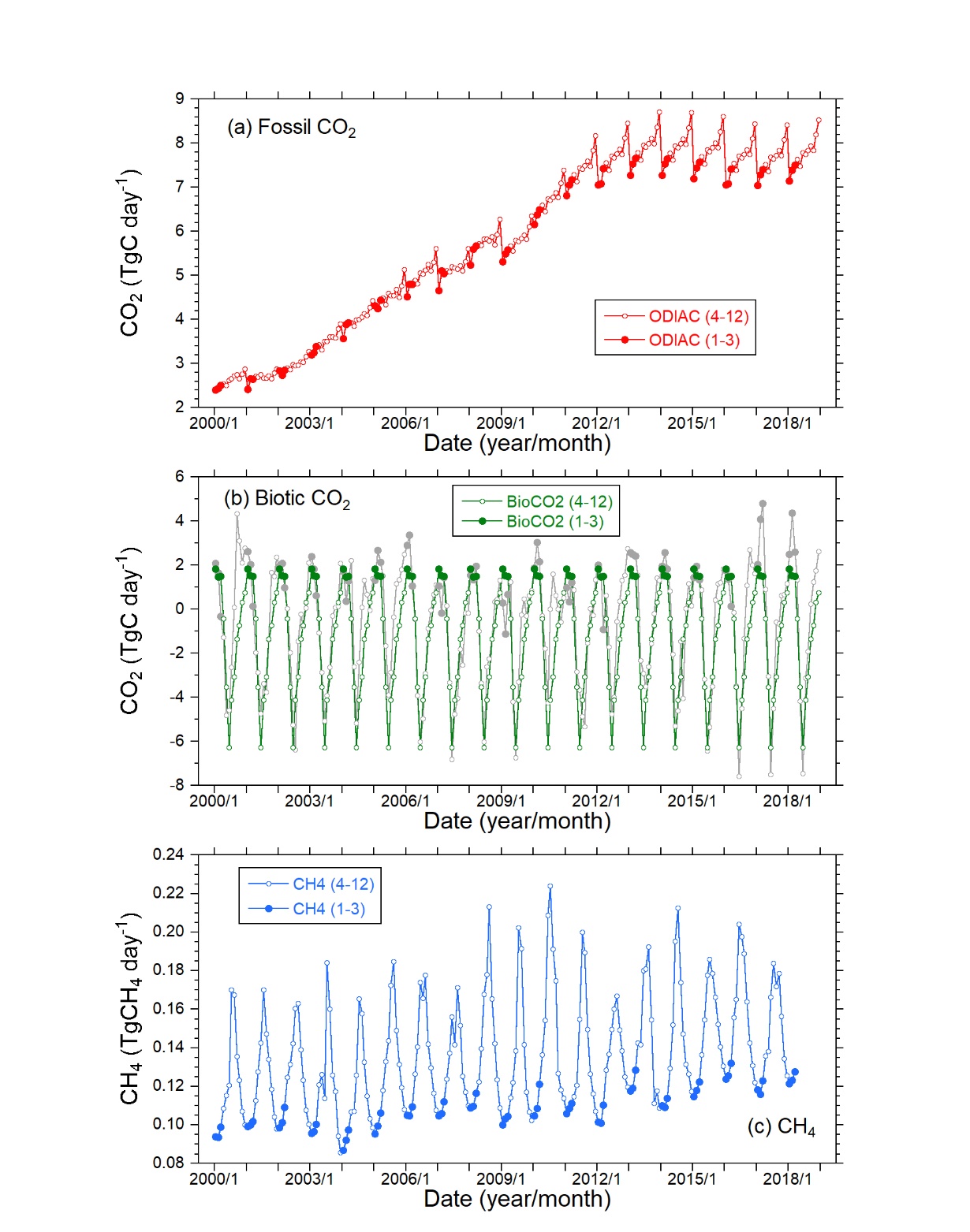


**Fig. S1.** Temporal changes in the monthly emissions of (a) FFCO_2_, (b) BioCO_2_, and (c) CH_4_ from China used in the simulation of this study. The closed symbols represent the emissions during January-March and the open symbols represent the emissions during April-December. Gray symbols in (b) represent monthly averages calculated from the inversion BioCO_2_ flux maps (see text).


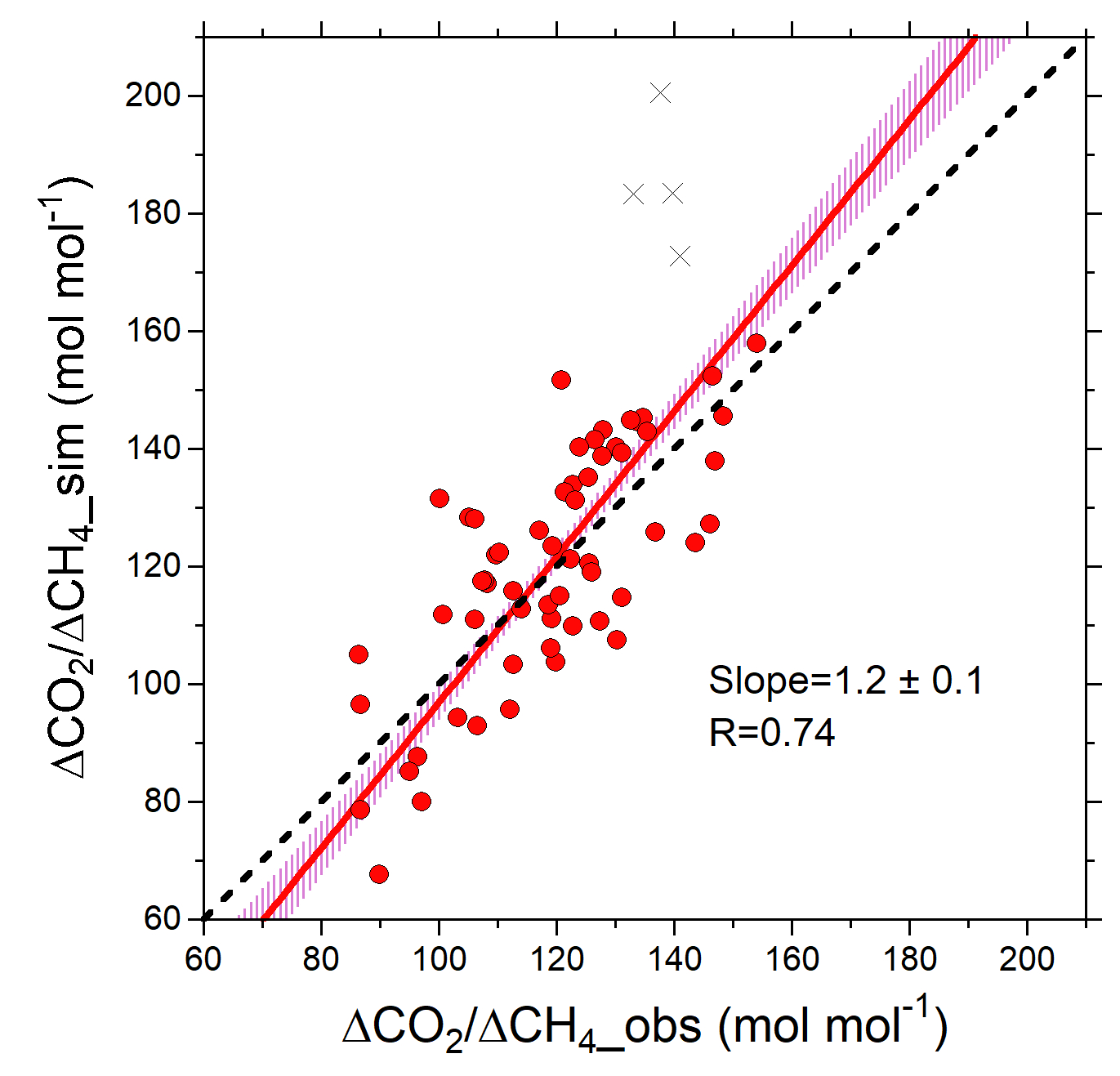


**Fig. S2.** Scatter plot of the relationship of the ΔCO_2_/ΔCH_4_ ratios (red circles) between the observation and simulation. The cross symbols represent the outliers of the simulated ΔCO_2_/ΔCH_4_ ratios. The black broken line represents the one-to-one line, and the red solid line and the vertical bars represent the linear regression line and the estimated uncertainties, respectively (1σ). The value of R represents the correlation coefficient of the plot.


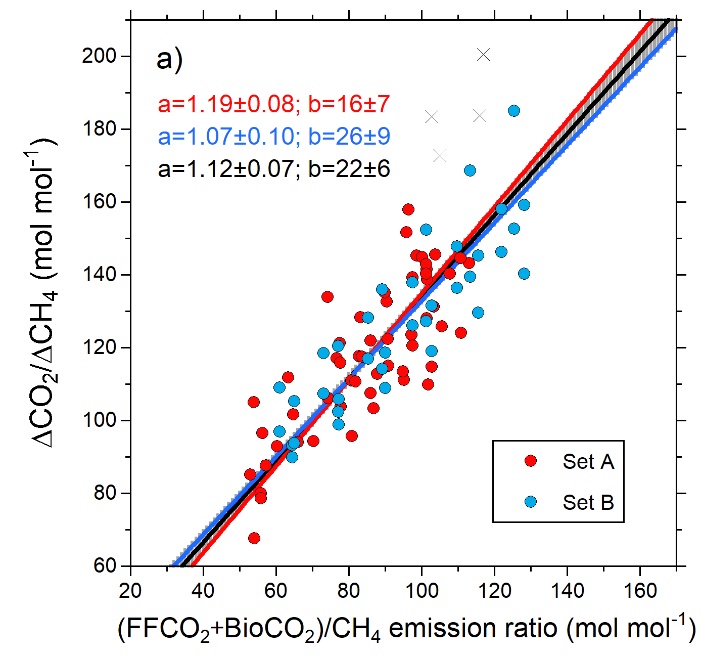

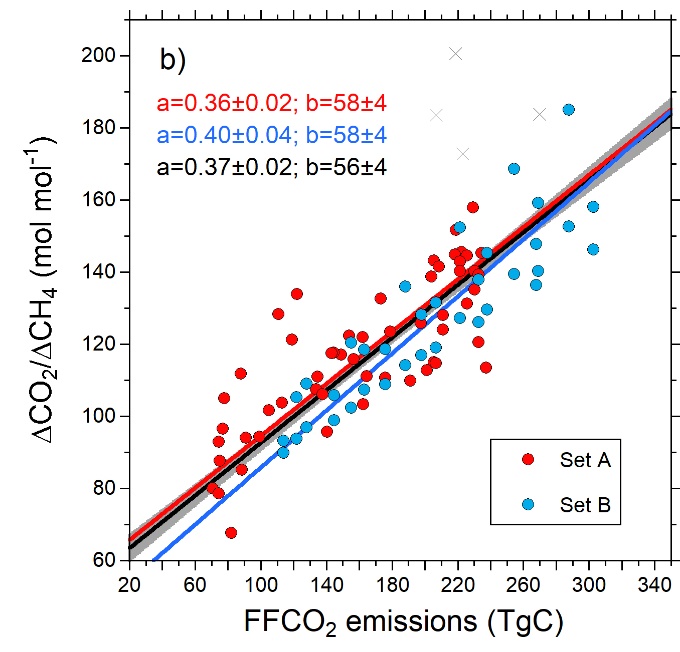


**Fig. S3.** Scatter plots of the simulated ΔCO_2_/ΔCH_4_ ratios for HAT to (a) the (FFCO_2_+BioCO_2_)/CH_4_ emission ratios and (b) the FFCO_2_ emissions in China. The red and blue circles represent the plots based on the FFCO_2_ emissions of Set A and Set B, respectively. The cross symbols are the outliers of the simulated ΔCO_2_/ΔCH_4_ ratios. The red, blue, and black lines represent linear regression lines for the plots based on Set A, Set B, and their combined set, respectively. The slopes and y-axis intercepts are shown in the figure. The gray vertical bars are estimated uncertainties (1σ) for the regression line based on the total FFCO_2_ data.


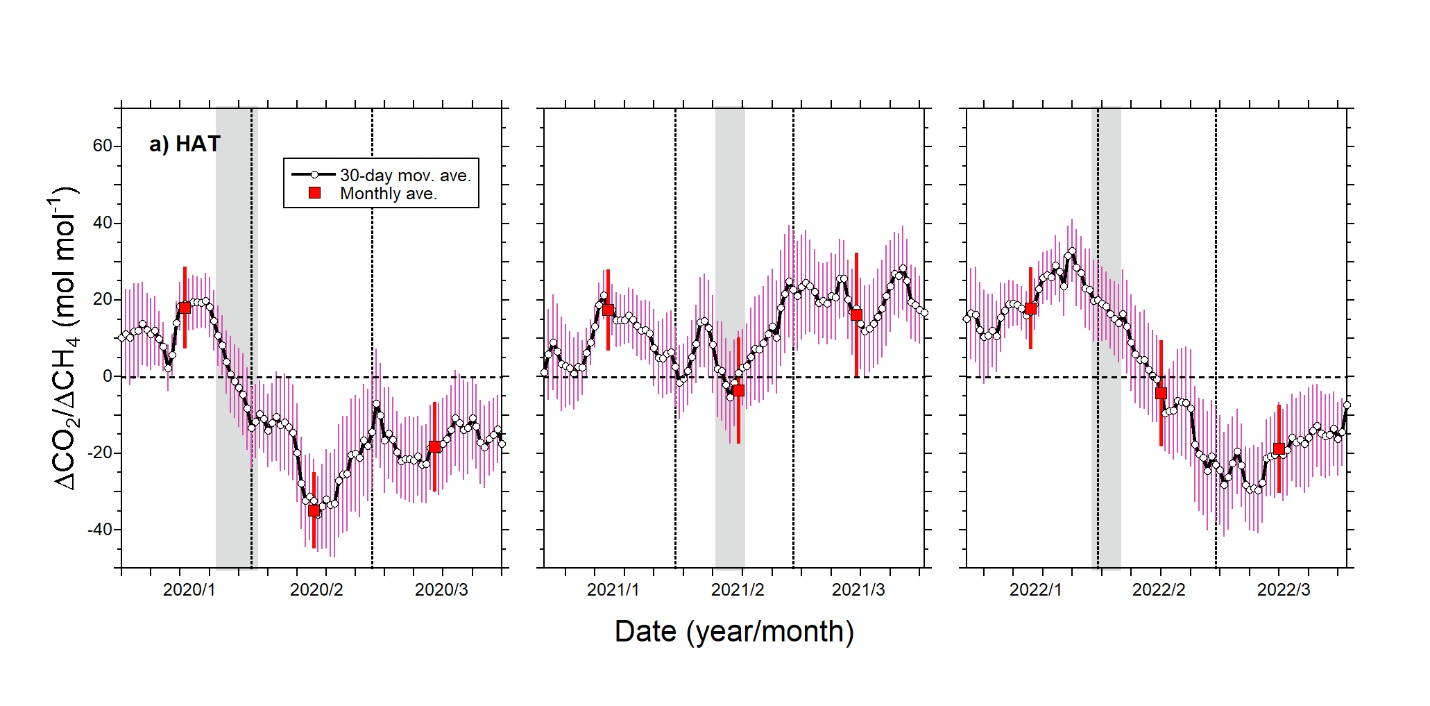


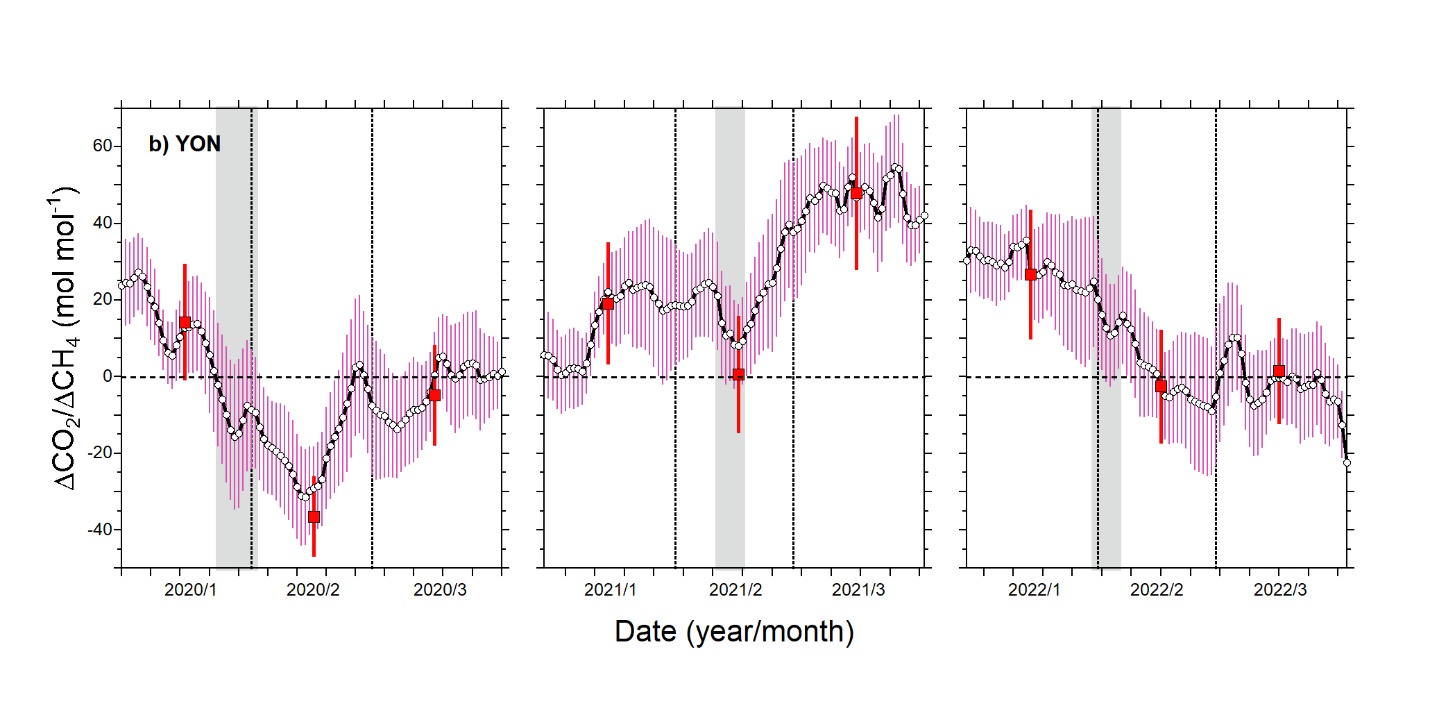


**Fig. S4.** Temporal changes of the FFCO_2_/CH_4_ emission ratio relative to the preceding 9-year (2011-2019) averages during the three-month period (January-March) in 2020, 2021, and 2022 for (a) HAT and (b) YON. The black open circles with lines and the red squares represent the 30-day moving averages and the monthly averages, respectively. The vertical bars represent the uncertainties. The gray shaded areas correspond to the Chinese New Year holidays.
